# A comparative study of machine learning models for automated detection and classification of retinal diseases in Ghana

**DOI:** 10.1371/journal.pone.0327743

**Published:** 2025-08-01

**Authors:** Gifty Duah, Eric Nyarko, Anani Lotsi

**Affiliations:** Department of Statistics and Actuarial Science, School of Physical and Mathematical Sciences, University of Ghana, Legon, Accra, Ghana; Eye Foundation Hospital/Eye Foundation Retina Institute, NIGERIA

## Abstract

**Introduction**:

Retinal diseases, a significant global health concern, often lead to severe vision impairment and blindness, resulting in substantial functional and social limitations. This study explored a novel goal of developing and comparing the performance of multiple state-of-the-art convolutional neural network (CNN) models for the automated detection and classification of retinal diseases using optical coherence tomography (OCT) images.

**Method**:

The study utilized several models, including DenseNet121, ResNet50, Inception V3, MobileNet, and OCT images obtained from the WATBORG Eye Clinic, to detect and classify multiple retinal diseases such as glaucoma, macular edema, posterior vitreous detachment (PVD), and normal eye cases. The preprocessing techniques employed included data augmentation, resizing, and one-hot encoding. We also used the Gaussian Process-based Bayesian Optimization (GPBBO) approach to fine-tune the hyperparameters. Model performance was evaluated using the F1-Score, precision, recall, and area under the curve (AUC).

**Result**:

All the CNN models evaluated in this study demonstrated a strong capability to detect and classify various retinal diseases with high accuracy. MobileNet achieved the highest accuracy at 96% and AUC of 0.975, closely followed by DenseNet121, which had 95% accuracy and an AUC of 0.963. Inception V3 and ResNet50, while not as high in accuracy, showed potential in specific contexts, with 83% and 79% accuracy, respectively.

**Conclusion**:

These results underscore the potential of advanced CNN models for diagnosing retinal diseases. With the exception of ResNet50, the other CNN models displayed accuracy levels that are comparable to other state-of-the-art deep learning models. Notably, MobileNet and DenseNet121 showed considerable promise for use in clinical settings, enabling healthcare practitioners to make rapid and accurate diagnoses of retinal diseases. Future research should focus on expanding datasets, integrating multi-modal data, exploring hybrid models, and validating these models in clinical environments to further enhance their performance and real-world applicability.

## Introduction

Retinal diseases such as diabetic retinopathy, age-related macular degeneration (AMD), glaucoma, and posterior vitreous detachment (PVD) present significant challenges to global health [1,2]. These conditions can lead to severe visual impairment and blindness, resulting in considerable functional and social limitations [1,3]. It is estimated that 2.2 billion people worldwide experience vision problems, with approximately 26.3 million individuals in Africa who have visual impairment and 5.9 million who are blind [4].

Preventing vision loss and blindness relies heavily on the early identification and accurate diagnosis of retinal disorders. Conventional diagnostic methods, while important, encounter significant challenges related to accessibility, cost, and diagnostic accuracy, particularly in resource-constrained settings where healthcare infrastructure is lacking [5]. Therefore, there is a critical need for a comprehensive approach that integrates artificial intelligence (AI), including machine learning (ML) algorithms and deep learning, with advanced imaging technologies. AI-driven systems can detect subtle anomalies or early indicators of disease that conventional examinations may overlook [6,7].

While previous studies have assessed traditional CNN models such as AlexNet, VGG16, and LeNet for retinal disease detection [8,9], these models often struggle with the intricate details of retinal images, which impacts their diagnostic performance. However, the emergence of modernized CNN models offers hope for more accurate and efficient diagnostic solutions, thereby overcoming these limitations and revolutionizing the detection of retinal diseases. For instance, a study on the automated detection of diabetic retinopathy using MobileNet, achieving 97.52% precision, 96.99% accuracy, and 98.54% recall. It also exhibits strong performance in specificity (97.23%), F1-score (93.8%), and AUC (0.927) [10]. Similarly, research on glaucoma detection utilized gray-scale fundus images with data augmentation to train a ResNet-50 CNN. Applied to the G1020 dataset, it achieved 98.48% accuracy, 99.30% sensitivity, 96.52% specificity, 97% AUC, and a 98% F1-score [11].

Additionally, another study explored capsule networks combined with contrast-limited adaptive histogram equalization (CLAHE) for classifying retinal optical coherence tomography (OCT) images, utilizing external data from institutions like the Shiley Eye Institute and the California Retinal Research Foundation [12].

In Ghana, there is a significant lack of studies that effectively utilize image analysis and ML techniques for diagnosing and detecting retinal diseases. Like many low- and middle-income countries (LMICs), Ghana faces considerable healthcare challenges, particularly in ophthalmology. Many individuals in the population remain undiagnosed or underdiagnosed for retinal diseases due to limited access to advanced diagnostic tools and specialized eye care professionals. This situation is further exacerbated by the rising prevalence of conditions such as diabetes, which increases the risk of diabetic retinopathy, along with an aging population that is more vulnerable to AMD and glaucoma [13,14]. A major limitation of existing research is the insufficient focus on local retinal disease patterns and the healthcare system relevant to Ghana. For instance, a study relied on external data [12] and findings from other countries, which may not accurately reflect the prevalence and healthcare needs of retinal diseases in Ghana. This gap underscores the need for locally sourced data that considers the country’s unique healthcare challenges and demographics. Moreover, a notable study primarily concentrated on a specific retinal disease [15], which limits the effectiveness of AI-driven diagnostic tools for patients who often present with multiple retinal conditions. To address this gap, the present study employs novel transfer learning techniques to develop models capable of classifying and detecting multiple retinal diseases from OCT images.

Although studies using CNNs for retinal disease detection abound in the literature, the present study explores a novel goal by developing and systematically comparing the performance of multiple CNN models—DenseNet121, ResNet50, Inception V3, and MobileNet—based on a local dataset. Furthermore, the CNN models are optimized using Gaussian Process-based Bayesian techniques to enhance diagnostic precision and efficiency. This integrated approach enhances model robustness, interpretability, and real-world clinical applicability. Enhancing local diagnostic practices through modernized CNNs will support better management of retinal diseases, contributing to broader eye health and vision preservation goals in Ghana and other LMICs while supporting the World Health Organization (WHO) 2030 targets on effective coverage of eye care [16].

### Research questions

The following are the main research questions of the study:

- How can modern CNN models (Inception V3, DenseNet121, MobileNet, and ResNet50) combined with Gaussian Process-based Bayesian Optimization effectively detect and classify multiple retinal diseases?
- How well do the pre-trained models perform in retinal disease-specific cases?

## Overview of existing literature

Significant advances have been made globally in the application of ML to detect and classify retinal diseases. Various models have shown promising results, particularly in segmentation, classification, and integrated approaches that combine both tasks. This section provides an overview of studies within these categories and emphasizes our unique multitask approach explored in the present research.

Segmentation of retinal OCT images is crucial for isolating anatomical structures, such as the retinal layers and the optic nerve head, which are essential for diagnosing retinal diseases. A study proposed a U-Net-based deep learning model for the segmentation of optic disc and optic cup regions from fundus images, which are critical indicators in glaucoma diagnosis [17]. Their approach integrated pre-trained convolutional backbones such as Inception V3, VGG19, and ResNet50 into an Attention U-Net framework, leveraging attention gates to enhance feature propagation across skip connections. The model was trained and evaluated using publicly available RIM-ONE and ACRIMA datasets, with preprocessing steps including contrast-limited adaptive histogram equalization (CLAHE) and median filtering applied to enhance image quality and reduce noise. Among the evaluated backbones, the ResNet50-based model achieved superior segmentation performance, attaining Dice coefficients of 99.60% for the optic disc and 97.75% for the optic cup on the RIM-ONE dataset. For classification, the Inception V3 backbone yielded a glaucoma detection accuracy of 98.79%. These results demonstrate the effectiveness of attention mechanisms in improving segmentation accuracy, as well as the potential of hybrid architectures for reliable glaucoma screening. Similarly, [18] introduced a U-Net model enhanced with hybrid attention mechanisms for automating sub-retinal layer segmentation in OCT images. This approach, which combines edge and spatial attention, achieved impressive performance metrics, including a Dice score of 94.99% and an Adjusted Rand Index (ARI) of 97.0%, outperforming existing methods. This model offers a reliable tool to advance AI-driven retinal diagnostics. Additionally, [19] utilized medical images and pre-trained residual convolutional networks (ResNet-50) to construct an early detection segmentation algorithm from digitized retinal fundus pictures.

Regarding classification, retinal OCT images have been analyzed to diagnose specific diseases such as diabetic macular edema (DME), glaucoma, or AMD. A study developed a multi-stage, multi-scale feature ensemble network for classifying OCT images, distinguishing between normal and abnormal retinal scans [20]. This model uses DenseNet as the backbone, combined in a pyramidal feature architecture, and achieved a binary (normal vs. abnormal) classification accuracy of 97.78%, with similarly high sensitivity and specificity rates. The authors emphasized the importance of transfer learning to improve classification performance, particularly for datasets with limited labeled images. To address the challenge of limited dataset size, [21] employed augmentation techniques. Their dataset contained only 69 images depicting vascular diseases and 55 healthy images. They trained a multilayer deep CNN for 10 epochs, achieving an accuracy of 88.4%. VGG-16 transfer learning has also been utilized to distinguish normal from abnormal OCT images, achieving over 90% accuracy and proving efficient for early-stage DME diagnosis [22]. In their study, VGG-16 served as a feature extractor, with the K-Nearest Neighbors (KNN) classifier yielding the highest accuracy.

Additionally, [23] explored deep learning algorithms for the early detection and timely treatment of ocular conditions using retinal fundus images. Their study utilized the retinal fundus multi-disease image dataset, which includes categories such as media haze, optic disc cupping, diabetic retinopathy (DR), and healthy eyes. They employed CNNs for automated diagnosis, applying preprocessing techniques such as data augmentation, cropping, resizing, and one-hot encoding to enhance model performance. Among the tested CNN models, a 12-layer CNN achieved accuracy rates of 89.81% for validation and 88.72% for testing, while a 20-layer CNN demonstrated slightly higher accuracy but exhibited overfitting. This study highlights the potential of CNNs in distinguishing multiple eye diseases from healthy images, offering a reliable and non-invasive diagnostic system for ocular conditions.

Some approaches integrate segmentation and classification into a sequential pipeline. For instance, [24] proposed a framework that first segments regions of interest (e.g., lesions and retinal layers) using a Mask R-CNN model and subsequently classifies the segmented regions into disease categories using a ResNet50 classifier. The study employed the OCT2017 dataset for AMD detection, reporting an overall classification accuracy of 95% and improved pathological feature localization. Additionally, utilizing a U-Net and transfer learning, [25] achieved state-of-the-art performance in segmenting and classifying diabetic retinopathy, suggesting potential for future refinements. Another study has indicated that a glaucoma screening method can be implemented in two stages [26]. First, optic disc segmentation is performed using the DeepLabv3+ architecture. Second, a pre-trained deep neural network is utilized for image classification. The study used ACRIMA dataset, achieving an impressive accuracy of 99.53% and an AUC of 99.98%.

Recent advancements in deep learning have significantly enhanced the detection and classification of retinal diseases, with techniques such as Attention U-Net demonstrating excellent performance in fundus image segmentation [27]. However, these methods primarily focus on segmentation and may not possess the efficiency and adaptability needed for multi-class disease classification.

A recent study introduced a federated epistasis detection framework (FedED-SegNAS) to identify disease-associated genetic variants while safeguarding data privacy across institutions [28]. This framework incorporates fuzzy logic into CNNs to enhance interpretability. In addition, it uses a search for neural architecture based on particle swarm optimization to improve model performance and communication efficiency during federated learning. FedED-SegNAS is particularly effective in multiinstitutional genomic research settings, where privacy and distributed data present significant challenges. In contrast, our study focuses on a unique approach to detecting and classifying retinal diseases using medical image data from an institution in a resource-limited setting. While FedED-SegNAS enhances interpretability through fuzzy logic, we employed CNNs in combination with Gradient-weighted Class Activation Mapping (Grad-CAM) and saliency map techniques to provide visual explanations for our CNN model’s predictions. This approach improves transparency and supports clinical decision-making by highlighting the regions of OCT images that most contribute to detection/classification outcomes.

Earlier research in Ghana utilized pulsed coupled neural networks (PCNN) and multilayer backpropagation neural networks (BPNN) for glaucoma detection [15]. However, our method represents a significant advancement. Unlike PCNN and BPNN, which relied on hand-made features and manual preprocessing [29], our use of CNNs facilitates automated hierarchical feature learning, making them more suitable for complex multiclass classification tasks. To further enhance performance, we applied Gaussian process-based Bayesian optimization to tune hyperparameters efficiently, outperforming traditional methods such as random or grid search [30]. Our model is designed to detect and classify multiple retinal conditions—including glaucoma, macular edema, PVD, and normal cases—while offering both accuracy and interpretability, specifically tailored to the needs of clinicians in low-resource settings.

## Methods

### Ethical considerations

The study was approved by the Ethics Committee of the College of Basic and Applied Sciences (ECBAS), University of Ghana (Reference No: ECBAS 052/23–24). The data were fully anonymized before being accessed, and the ECBAS waived the requirement for informed consent.

### Data gathering

**Fig 1:**
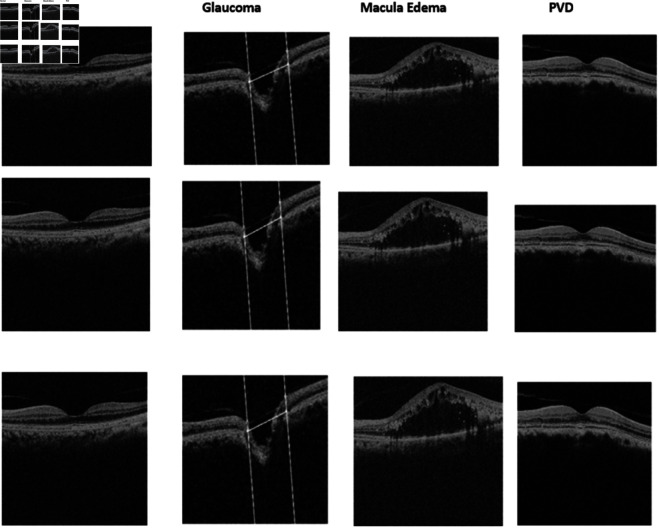
Sample retinal OCT images.

A comprehensive dataset of retinal images was obtained from the WATBORG Eye Clinic in Ghana on May 24, 2024. This clinic is renowned for its advanced facilities and extensive patient database, making it an ideal setting for a detailed investigation into the detection and classification of retinal diseases. Focusing on data from this clinic ensured access to high-quality information, essential for applying and evaluating deep learning methods in retinal disease analysis. The dataset includes OCT images crucial for diagnosing and monitoring various retinal conditions, including glaucoma, macular edema, PVD, and healthy eye conditions. While this dataset provides valuable real-world clinical data, we relied on data from a single health facility in Ghana, which may not fully represent the broader Ghanaian population or other regions with different demographic or epidemiological characteristics. Fig 1 shows a sample of these images.

The data collection process was designed to guarantee the quality and integrity of the final dataset. We compiled a comprehensive list of unique identifiers for individual patients and leveraged the RAND() function in Microsoft Excel to randomly select participants. This method ensured that each patient had an equal chance of inclusion in the study, thereby minimizing potential bias in the selection process and enhancing the credibility of the final dataset. It is important to note that this study included patients with a confirmed diagnosis of one of four retinal conditions: glaucoma, macular edema, PVD, and normal eye status. To achieve the highest level of accuracy, these clinical diagnoses were validated by experienced ophthalmologists at the WATBORG Eye Clinic. We excluded cases with ambiguous diagnoses or inadequate imaging quality from the study, which helps ensure the accuracy and reliability of the dataset. In total, 889 images were used in this study: 498 images were allocated for training, 266 for testing, and 125 for validation. Table 1 presents the distribution of images within the dataset.

**Table 1:** Distribution of the dataset.

	Glaucoma	Macula Edema	PVD	Normal	Total
Training	145	72	112	167	498
Test	78	39	59	90	266
Validation	37	19	29	42	125
Total	260	130	200	299	889

### Research design

This study conducted a comprehensive analysis to significantly enhance the accuracy and efficiency of detecting and classifying multiple retinal diseases using advanced CNN models. It began with inputting retinal OCT images into training and testing datasets. Inception V3, MobileNet, DenseNet121, and ResNet50 models were utilized for feature extraction and classification of these images. The results from these models were combined into an ensemble model to distinguish between normal and unhealthy retinal conditions, with unhealthy conditions further classified into glaucoma, macular edema, and PVD. The findings of this study hold significant implications for retinal disease detection and the advancement of medical AI in ophthalmology.

A robust hold-out validation approach was used to evaluate model performance. The dataset was split into training and test sets, with part of the training set further allocated for validation. The training set was used to fit the model, while the validation set helped fine-tune the hyperparameters. Bayesian optimization was applied to explore the hyperparameter space efficiently, maximizing the model’s performance. The objective was to distinguish between normal and diseased retinal conditions, further classifying diseases into glaucoma, macular edema, and PVD. This approach emphasizes the study’s relevance to practical applications in retinal disease detection.

Model performance was evaluated using key metrics such as precision, recall, accuracy, F1-score, area under the curve (AUC), and confusion matrices. Particular attention was given to recall, ensuring the models accurately identified disease cases, minimizing the likelihood of misdiagnosis. The study’s use of modern deep learning techniques, hold-out validation, and Bayesian optimization for hyperparameter tuning contributed to the system’s robustness and high accuracy in detecting retinal diseases, underscoring the cutting-edge nature of the research.

All the analysis were conducted using Python 3.8 and several libraries for deep learning, data processing, and evaluation. The core deep learning model was implemented with TensorFlow (version 2.x), utilizing the pre-trained MobileNet architecture for transfer learning. NumPy was used for numerical computations and array manipulations, while the ImageDataGenerator from TensorFlow facilitated data augmentation and preprocessing. We computed metrics such as classification reports, confusion matrices, and ROC-AUC scores using scikit-learn. Matplotlib was employed to visualize metrics and results, and hyperopt was used to optimize hyperparameters through Bayesian methods. Additionally, itertools supported the creation of combinations and iteration over model parameters. It is worth noting that the trials were conducted on a 64-bit version of the Windows 10 operating system. The system was equipped with an Intel Core i5 7th Generation CPU, 8 GB of RAM, and a storage capacity of 237 GB, which provided sufficient computational resources for the analysis.

The following details the key research design approach adopted in this study:

#### Data input.

The first stage involved inputting images from the retinal OCT scan dataset. An image can be expressed mathematically as a matrix **I** of size m×n, where *m* is the number of rows (height of the image), *n* the number of columns (width of the image), and *I*(*i*,*j*) represents the pixel value at the *i*-th row and *j*-th column. For a grayscale image, *I* contains values that represent the intensity of each pixel, typically ranging from 0 (black) to 255 (white) in an 8-bit image [31]. For a color image, each pixel is typically represented by three values corresponding to the Red, Green, and Blue channels, often stored as three separate matrices or a 3-dimensional matrix [32]. A grayscale image matrix is given by


$$I=(\begin{array}{cccc} I(1,1) & I(1,2) & \cdots & I(1,n)\\ I(2,1) & I(2,2) & \cdots & I(2,n)\\ \vdots & \vdots & \ddots & \vdots \\ I(m,1) & I(m,2) & \cdots & I(m,n) \end{array}),$$

where as before, each element *I*(*i*,*j*) represents the pixel’s intensity at position (*i*,*j*) in the image.

#### Pre-processing.

Pre-processing is essential for enhancing and improving the quality of image visualization, and it significantly influences the success and accuracy of subsequent stages in our proposed method. Medical images often contain extra content that can compromise clarity, leading to suboptimal visualization and less accurate results. During the pre-processing stage, we implemented several techniques to improve model efficiency. Data enhancement, cropping, resizing, data set splitting, and one-hot encoding [23,33] were applied to optimize the data set.

We employed noise reduction filters, such as Gaussian blur and median filtering, and applied contrast enhancement techniques [34–36] to clarify and emphasize key features to enhance image quality. Pixel value normalization was conducted to scale pixel intensities to a common range, typically 0 to 1, promoting consistency across images. Additionally, edge detection algorithms [37–41] were employed to identify and highlight object boundaries, facilitating effective segmentation in subsequent stages.

#### Data augmentation and splitting.

We implemented various augmentation techniques to guarantee that our dataset encompasses diverse and balanced data. These techniques were employed to enrich the variability of the dataset, thereby reducing the risk of overfitting, which occurs when the model performs well on the training data but poorly on new, unseen data. By applying augmentation methods such as rotation, flipping, and translation, we aimed to enhance our modernized CNN models’ robustness and generalization capability. Specifically, images were rotated by 90° and 270°, centered using the following equations:

$$x^{\prime }=x\cos\theta -y\sin\theta$$ (1)

$$y^{\prime }=x\sin\theta +y\cos\theta ,$$ (2)

where θ is the rotation angle, (*x*,*y*) represents the original coordinates of a pixel in the image, and $\left(x^{\prime },y^{\prime }\right)$ represents the new coordinates of the pixel after rotation. Moreover, images were flipped both vertically and horizontally as

$$x^{\prime }=x$$ (3)

$$y^{\prime }=\text{ymax}-y,$$ (4)

where ymax is the maximum y-coordinate of the image. Finally, images were translated by shifting marginally in the *X*-axis and *Y*-axis directions

$$x^{\prime }=x+x_{T}$$ (5)

$$y^{\prime }=y+y_{T}$$ (6)

where *x*_*T*_ is the translation shift in the horizontal *X*-axis direction, *y*_*T*_ is the translation shift in the vertical *Y*-axis direction.

Here, we use on-the-fly augmentation due to its efficiency and ability to reflect real-world data variability better. On-the-fly augmentation involves applying augmentation techniques in real time during training, enhancing data variability without storing augmented images. This method is efficient and reflects real-world data conditions [39]. The dataset was split into training, testing, and validation sets. It was split in the common ratio: 70% of the augmented dataset for training, 15% for validation, and 15% reserved for testing.

#### Feature extraction and classification.

Feature extraction and classification are two different processes in ML. Common features for classifying melanoma include asymmetry, border irregularity, color, and texture. This work uses CNNs for classification and modernized pre-trained models, including MobileNet, Inception V3, DenseNet121, and ResNet50. These models are modern and sophisticated, offering a varied assessment of CNN architectures in retinal disease classification. They are well known for working well on various computer vision applications, such as classifying images.

## Convolutional neural network model

CNNs are a class of deep neural networks specifically designed for computer vision and image processing tasks. They have revolutionized applications such as image recognition and object detection. Unlike traditional artificial neural networks, which typically consist of a single input layer, hidden layers, and an output layer [40], CNNs, as similarly noted, incorporate specialized layers to handle spatial hierarchies in images.

In CNN, the input image ${\text{x}}^{1}$ undergoes processing through multiple layers, denoted as $x^{2},x^{3},\ldots ,x^{L}$, where each layer applies different transformations. The network architecture comprises convolutional layers, pooling layers, activation functions, and fully connected layers. Specifically, the convolutional operation is defined as

$$A_{j}=f\left(\sum_{i=1}^{N}I_{i}\cdot K_{ij}+B_{j}\right),$$ (7)

where *I*_*i*_ represents the input elements, *K*_*ij*_ is the kernel matrix, *B*_*j*_ is the bias term, and the activation function *f*(.) uses filters (or kernels) to extract features from the input image. Each filter performs convolution operations to produce feature maps, highlighting different image aspects. Moreover, the pooling layer reduces the dimensionality of feature maps while preserving essential information. Max pooling [41] is commonly used, which extracts the maximum value from each sub-region of the feature map, which can be formulated as


$$y_{l+1,j_{l+1},d}=\max_{0\leq i<H,0\leq j<W}x_{l,(i_{l+1}\times H+i),(j_{l+1}\times W+j),d},$$

where $y_{l+1,j_{l+1},d}$ is the output value after pooling at layer l+1, for position *j*_*l* + 1_ and depth *d*, *x*_*l*_ represents the input feature map at layer *l* (before pooling). Here *H*,*W* are the height and width of the pooling window, respectively, max(·) is the max function, which selects the highest value in each region of the feature map and $i_{l+1}\;\times \;H\;+\;i,j_{l+1}\;\times \;W\;+\;j$ are the coordinates used to determine the position in the input feature map. The activation functions [42], however, introduce non-linearity into the model, allowing CNNs to learn complex patterns. The widely used Rectified Linear Unit (ReLU) is given by

F(X)=max(0,x), (8)

where *x* represents the input to the activation function, typically the output of a neuron before activation.

Now, the fully connected layer serves as the classifier, making final predictions based on high-level features extracted by previous layers. The softmax function is given by

$$z^{k}=\frac{e^{z_{j}}}{\sum_{k=1}^{K}e^{z_{k}}}\;\text{for }j\in \{1,\ldots ,K\},$$ (9)

where ${\text{z}}^{\text{k}}$ represents the probability of the input belonging to class *k*, and $e^{z_{k}}$ is the exponential of the raw score for class *k* is commonly employed to output probabilities for multi-class classification.

As was similarly noted, a range of modernized pre-trained models, such as MobileNet, Inception V3, DenseNet121, and ResNet50, were used for feature extraction and classification of OCT images. Specifically, ResNet50 introduces a novel architecture with 48 convolutional layers, 1 max-pooling layer, and 1 average-pooling layer. It is distinguished by its shortcut connections and residual functions, which address training challenges in very deep networks. These connections allow the model to skip layers, reducing training errors and facilitating the learning process [43].

Additionally, Inception V3, an advanced iteration of the Inception models, consists of 48 layers and is part of the GoogleNet family. It incorporates factorized convolutions, auxiliary classifiers, and label smoothing. These enhancements contribute to its robust performance on large datasets, making it suitable for various computer vision tasks [44].

DenseNet121, on the other hand, features dense connectivity, where each layer receives inputs from all previous layers and contributes to subsequent layers. It utilizes bottleneck and transition layers to manage computational costs and parameters efficiently. The architecture ends with global average pooling and a fully connected layer. DenseNet121’s dense connectivity promotes feature reuse, enhancing its performance in image classification and object detection tasks [45].

Moreover, MobileNet, developed by Google, is designed for mobile and embedded vision applications. It employs depthwise separable convolutions to reduce the computational cost and number of parameters. Variants include MobileNet V1, V2, and V3, each incorporating improvements such as inverted residuals and the squeeze-and-excitation module. MobileNet models are ideal for deployment on resource-constrained devices, balancing efficiency and accuracy [46].

### Tuning of hyperparameters

Deep learning models necessitate tuning of hyperparameters, such as learning rate, number of layers, and number of units per layer, before the training can commence. Hyperparameter optimization involves adjusting these settings to enhance the performance of a neural network. This process includes modifying architectural aspects like the size of hidden layers, dropout rates, choice of activation functions, weight initialization, and training parameters such as learning rates, momentum, batch sizes, and the number of training epochs. Hyperparameter tuning aims to find the most effective configurations for these settings, minimize the loss function, and enhance overall model performance.

Various methods are available for hyperparameter optimization, each with unique approaches and benefits. These methods include manual exploration, grid search, random search, and Bayesian optimization. In this study, we employed Gaussian process-based Bayesian optimization. Hyperparameter optimization can be formulated as

$$X_{\text{opt}}=\arg\max_{x\in X}f(x),$$ (10)

where *x* represents the hyperparameters, *X* is the feasible set (domain), and *f*(*x*) is the objective function, often involving model performance evaluation metrics.

#### Gaussian process-based Bayesian optimization.

Bayesian optimization is an efficient algorithm for addressing optimization problems. It combines prior knowledge about the unknown function with sampled information to update insights into the function’s distribution, using Bayes’ theorem [47]. Given evidence *E*, the posterior probability P(M|E) of a model *M* is proportional to the likelihood P(E|M) of observing *E* given model *M*, multiplied by the prior probability *P*(*M*):

P(M|E)∝P(E|M)P(M). (11)

Bayesian optimization consists of two main components: a probabilistic (surrogate) model for approximating the objective function and an acquisition function that navigates the exploration versus exploitation trade-off. In this study, a Gaussian process is used for the probabilistic model.

#### Estimation of the posterior function.

Given a set of hyperparameters λ, the posterior probability P(m|λ) of a model *m* is proportional to the likelihood P(λ|m). To obtain the posterior distribution, bayesian optimization combines the prior distribution of *f*(*x*) with sample information. The posterior information is then used to maximize *f*(*x*) according to a criterion.

Assuming the prior distribution *f*(*x*) follows a Gaussian process:

$$f(x)\sim GP(m(x),k(x,x^{\prime }))$$ (12)

where

$$k(x,x^{\prime })=\exp\left(-\frac{1}{2}\|x-x^{\prime }{\|}^{2}\right)$$ (13)

and


m(x)=0.

In estimating the posterior function, we consider *t* observations as the training set $D_{1:t}=\{f(x_{n})|n=1,\ldots ,t\}$. We assume *f* is drawn from a multivariate normal distribution:

f~𝒩(0,k), (14)

where *k* is given by


$$[\begin{array}{cccc} k(x_{1},x_{1}) & k(x_{1},x_{2}) & \cdots & k(x_{1},x_{t})\\ k(x_{2},x_{1}) & k(x_{2},x_{2}) & \cdots & k(x_{2},x_{t})\\ \vdots & \vdots & \ddots & \vdots \\ k(x_{t},x_{1}) & k(x_{t},x_{2}) & \cdots & k(x_{t},x_{t}) \end{array}].$$

Each element in *k* is computed by the kernel function $k(x_{i},x_{j})$, which measures the degree of approximation between two samples.

For a new sample point *x*_*t* + 1_, the function value *f*_*t* + 1_ follows a t+1 dimensional normal distribution:

$$[\begin{array}{c} f_{1:t}\\ f_{t+1} \end{array}]\sim 𝒩(0,k),$$ (15)

where $\left[k(x_{t+1},x_{1}),k(x_{t+1},x_{2}),\ldots ,k(x_{t+1},x_{t})\right]$ represents the covariance vector.

#### Acquisition function.

After estimating the posterior function, Bayesian optimization uses an acquisition function *u* to determine the maximum of the function *f*. Typically, a high value of the acquisition function indicates a large value of the objective function. Thus, maximizing the acquisition function is equivalent to maximizing *f*:

$$x^{+}=\arg\max_{x\in A}u(x|D).$$ (16)

Common acquisition functions include Probability of Improvement (PI), Expected Improvement (EI), and GP Upper Confidence Bound (GP-UCB). Specifically, the PI function explores near the current optimal value to find points most likely to surpass the current optimum. The expression for PI is:

$$PI(x)=P\left[f(x)\geq f(x^{+})\right]=\phi \left(\frac{\mu (x)-f(x^{+})}{\sigma (x)}\right).$$ (17)

To address the limitation of PI, an ϵ parameter is introduced

$$PI(x)=P\left[f(x)\geq f(x^{+})+\epsilon \right]=\phi \left(\frac{\mu (x)-f(x^{+})-\epsilon }{\sigma (x)}\right).$$ (18)

Moreover, the EI quantifies the expected gain of the objective function when exploring the current optimum’s vicinity. If the improvement is less than the expected value, the current optimal point may be a local optimum. The improvement *I*(*x*) is given by

$$I(x)=\max\{0,f_{t+1}(x)-f(x^{+})\}.$$ (19)

The expected improvement is:

$$E(I)=\int_{-\infty }^{+\infty }If(I)\;dI$$ (20)

$$=\int_{I=0}^{+\infty }I\frac{1}{\sqrt{2\pi \sigma^{2}(x)}}\exp\left(-\frac{(\mu (x)-f(x^{+})-I{)}^{2}}{2\sigma^{2}(x)}\right)\;dI$$ (21)

=σ(x)[ZΦ(Z)+Φ(Z)], (22)

where $Z=\frac{\mu (x)-f(x^{+})}{\sigma (x)}$.

Again, the GP-UCB acquisition function balances exploration and exploitation. It is defined as

UCB(x)=μ(x)+κσ(x), (23)

where κ controls the trade-off between exploring areas of high uncertainty and exploiting areas with high predicted mean.

Table 2 shows the CNN architectural model configuration with their names and corresponding parameters.

**Table 2:** Model parameters.

Name	Parameter
Input	OCT images
Image Size	224 × 224 × 3
Batch Size	32
Activation Function	ReLU, Softmax
Number of Epochs	5
Dropout	50%
Optimization Function	Adam Optimizer
Loss Function	Categorical Cross-entropy
Fine-tuning of hyperparameters	Gaussian process-based Bayesian optimization

### Performance analysis method

This study used a comprehensive approach to identifying and classifying retinal diseases using a set of evaluation performance metrics. These metrics included the accuracy score (ACU), precision (PRE), recall (RE), and F1-score. By employing these diverse metrics, the study aimed to ensure a balanced and thorough assessment of the diagnostic performance. The analytical methods utilized in this study provided robust and dependable estimations, contributing to the reliability of the findings. These methods of formulation can be stated as:

$$ACU==\frac{TP+TN}{TP+TN+FP+FN}.\;RE==\frac{TP}{TP+FN}.\;PRE==\frac{TP}{TP+FP}.\;F1-score==\frac{2\times PRE\times RE}{PRE+RE}.\;TPR==Recall=\frac{TP}{TP+FN}.\;FPR==1-Specificity=\frac{FP}{FP+TN}.$$ (24)

All methods use the terms TP, TN, FP, and FN, representing true positive, true negative, false positive, and false negative, respectively. TP signifies correctly recognized positive aspects, while TN signifies properly detected negative instances. On the other hand, FP indicates the misclassification of negative occurrences as positive, and FN indicates the incorrect identification of positive examples as negative [48]. Another important performance measure used in this study to evaluate models is the receiver operating characteristic (ROC) curve. This curve plots the True Positive Rate (TPR) against the False Positive Rate (FPR) at different threshold values. The Area Under the ROC curve (AUC) measures the overall ability of the model to distinguish between classes.

Moreover, the confusion matrix presented in Table 3 is another crucial tool employed in this study in classification tasks as it comprehensively summarizes a classifier’s performance. Each cell in the table shows the count of true and false classifications, both positive and negative. This matrix helps evaluate the accuracy of predictions by comparing them to the true values. The confusion matrix is represented as

**Table 3:** Confusion matrix.

	Positive	Negative
Positive	True Positive (TP)	False Positive (FP)
Negative	False Negative (FN)	True Negative (TN)

Also, here, TP indicates correctly predicted positive cases, FP indicates incorrectly predicted positive cases, TN indicates correctly predicted negative cases, and FN indicates incorrectly predicted negative cases.

## Results

We examined the performance of the pre-trained CNN models used in this study: Inception V3, DenseNet121, MobileNet, and ResNet50. We tuned the hyperparameters for each model using Bayesian optimization, and the final values are presented in Table 4. All models utilized the highly efficient Adam optimizer. The optimized learning rates were 0.0004 for Inception V3, 0.0005 for DenseNet121 and MobileNet, and 0.0008 for ResNet50. ReLU activation was applied to the hidden layers, while the Softmax function was used for the output layers. The number of neurons was set to 512 for Inception V3 and ResNet50, 256 for DenseNet121, and 1024 for MobileNet. A consistent batch size of 32 was employed across all models. These hyperparameter values, optimized through Bayesian optimization, resulted in improved model performance for the classification task.

**Table 4:** Hyperparameters with tuned values.

Hyperparameters	Inception V3	DenseNet121	MobileNet	ResNet50
Optimizer	Adam	Adam	Adam	Adam
Learning Rate	0.0004	0.0005	0.0005	0.0008
Activation (Hidden)	ReLU	ReLU	ReLU	ReLU
Activation (Output)	Softmax	Softmax	Softmax	Softmax
Number of Neurons	512	256	1024	512
Batch Size	32	32	32	32

### Overall performance of the CNN models

The comparative analysis of the modernized CNN models reveals several key insights. As shown in Table 5, modernized CNN models have introduced significant advancements in performance and efficiency. Inception V3, with an accuracy of 83%, a precision of 90%, and an F1-score of 84%, demonstrates robust performance. It also achieves an exceptional ROC AUC of 99% while maintaining a relatively short training time of 6 minutes. This efficiency highlights its effective use of computational resources.

**Table 5:** Comparison of modernized CNN models.

Model Name	Accuracy	Precision	Recall	F1-score	ROC AUC	Training Time
Inception V3	83%	90%	82%	84%	99%	6 mins
DenseNet121	95%	95%	95%	95%	99%	7 mins
MobileNet	96%	97%	97%	97%	99%	5 mins
ResNet50	61%	63%	65%	58%	88%	6 mins

DenseNet121 has the highest metrics among the models analyzed. It achieves an impressive accuracy of 95%, precision of 95%, recall of 95%, and an F1-score of 95%. The ROC AUC for DenseNet121 is also excellent, at 99%. Despite its superior performance, the model requires a training time of 7 minutes, which is efficient considering its high accuracy level and detail.

MobileNet also stands out with the highest accuracy, 96% precision, 97% recall, and 97% F1-score. It also has the shortest training time, 5 minutes, making it highly efficient. Its lightweight architecture contributes to its quick training while maintaining exceptional performance across all metrics.

On the other hand, ResNet50, although it has a competitive training time of 6 minutes, demonstrates relatively lower performance compared to other modernized models. It records an accuracy of 61%, precision of 63%, recall of 65%, and an F1-score of 58%. The ROC AUC for ResNet50 is 88%, indicating that while it performs reasonably well, it is less effective for the tasks evaluated compared to the more modern architectures.

The performance of these models can be attributed to their deep architectures and efficient feature extraction capabilities. DenseNet121’s dense connectivity and MobileNet’s lightweight design contribute to their strong performance. DenseNet121’s ability to retain gradient flow throughout the network enhances its learning capability, making it highly effective for complex classification tasks. MobileNet, designed for efficiency, balances model performance with computational constraints, making it suitable for practical deployment in real-world scenarios.

### Disease-wise comparison of modernized CNN model performance

Table 6 compares the performance of each model across various retinal conditions, including glaucoma, normal eye, macular edema, and PVD. It highlights their unique strengths and weaknesses in disease classification based on precision, recall, and F1-score.

**Table 6:** Disease-wise comparison of model performance.

Disease	Model	Precision (%)	Recall (%)	F1-Score (%)
Glaucoma	Inception V3	High (91)	High (95)	High (93)
	DenseNet121	High (88)	High (99)	High (93)
	ResNet50	High (88)	High (97)	High (93)
	MobileNet	High (89)	High (99)	High (93)
Normal Eye	Inception V3	Low (69)	High (92)	Moderate (79)
	DenseNet121	High (99)	High (86)	High (92)
	ResNet50	Low (40)	Low (0.07)	Low (0.11)
	MobileNet	High (89)	High (99)	High (94)
Macular Edema	Inception V3	High (100)	High (92)	High (96)
	DenseNet121	High (97)	High (100)	High (99)
	ResNet50	Moderate (81)	Low (56)	Low (67)
	MobileNet	High (100)	High (100)	High (100)
PVD	Inception V3	High (100)	Low (49)	Low (66)
	DenseNet121	High (98)	High (100)	High (99)
	ResNet50	Low (43)	High (100)	Low (60)
	MobileNet	High (100)	High (100)	High (100)

For the classification of glaucoma, the Inception V3 model demonstrates superior performance, surpassing other models in terms of precision (91%), recall (95%) and F1-score (93%). This indicates that Inception V3 is adept at correctly identifying glaucoma cases while minimizing false positives and negatives. The architecture of this model, which effectively captures intricate features at multiple scales, likely contributes to its exceptional performance in detecting glaucoma. The DenseNet121 model also performed commendably well with high precision (88%), recall (99%), and F1-score (93%). The ResNet50 model shows high precision (88%), recall (97%), and F1-score (93%) but lagged slightly behind the others. MobileNet also demonstrated high performance with precision (89%), recall (99%), and F1-score (93%), comparable to DenseNet121 and Inception V3. Inception V3 showed high precision (100%), low recall (49%), and low F1-score (66%).

In terms of classifying normal eye conditions, the DenseNet121 model outperformed all other models with highest precision (99%), recall (86%), and F1-score (92%). This indicates that DenseNet121 is highly effective at distinguishing healthy eyes from those with pathological conditions. MobileNet, however, showed high precision (89%), recall (99%), and F1-score (94%), making it a strong contender alongside DenseNet121.

MobileNet achieved perfect scores with precision (100%), recall (100%), and F1-score (100%) when classifying macular edema, underscoring its robustness and adaptability in this domain. Both the Inception V3 and DenseNet121 models demonstrated excellent performance. Inception V3 had high precision (100%), recall (92%), and F1-score (96%), while DenseNet121 achieved high precision (97%) and F1-score (99%). These models’ high precision and recall values indicate their effectiveness in correctly identifying macular edema cases while minimizing misclassifications. The architectural strengths of Inception V3 in capturing multi-scale features and DenseNet121’s ability to maintain information flow across layers contribute to their superior performance in macular edema classification. ResNet50 models, although useful, did not achieve the same level of effectiveness as Inception V3 and DenseNet121 for this specific disease.

In the classification of PVD, ResNet50 showed low precision (43%), high recall (100%), and low F1-score (60%). MobileNet achieved perfect scores with precision (100%), recall (100%), and F1-score (100%), underscoring its robustness and adaptability in this domain. DenseNet121 again outperformed the other models, achieving high precision (98%), recall (100%), and an F1-score of 99%. This model’s effective feature propagation and resilience to gradient vanishing contribute to its robust performance in identifying PVD cases. While providing valuable classification capabilities, other models, including Inception V3 and ResNet50, did not match DenseNet121’s effectiveness in PVD classification. This underscores DenseNet121’s versatility and robustness across different retinal diseases.

### Confusion matrix for all the CNN models

The analysis of the confusion matrices provides comprehensive insights into each model’s classification performance across the different retinal diseases. As shown in Table 7, DenseNet121 demonstrated excellent performance across all categories. It achieved 77 true positives and only 1 false positive for glaucoma, 39 true positives with no false negatives for macular edema, 77 true positives with 11 false negatives for normal eye conditions, and 59 true positives with no false positives or false negatives for PVD. This model’s performance is characterized by minimal misclassifications, reflecting its robust feature extraction and classification capabilities.

**Table 7:** Confusion matrix for Dense121.

	Glaucoma	Macula Edema	Normal	PVD
Glaucoma	77	0	1	0
Macula Edema	0	39	0	0
Normal	11	1	77	1
PVD	0	0	0	59

The Inception V3 model presented in Table 8 showed strong performance in glaucoma and normal eye classification, with 74 true positives and 4 false negatives for glaucoma and 83 true positives with 7 false negatives for normal eye conditions. It had 36 true positives and 3 false negatives for macular edema. However, Inception V3 struggled with PVD detection, where it correctly identified only 29 cases and had 30 false negatives. This indicates that while Inception V3 is effective in certain areas, it has limitations in others.

**Table 8:** Confusion matrix for Inception V3.

	Glaucoma	Macula Edema	Normal	PVD
Glaucoma	74	0	4	0
Macula Edema	0	36	3	0
Normal	7	0	83	0
PVD	0	0	30	29

In Table 9, MobileNet performed exceptionally well across all categories, demonstrating high precision and recall. It achieved 77 true positives and only 1 false positive for glaucoma, 39 true positives with no false negatives for macular edema, and 80 true positives with 10 false negatives for normal eye conditions. For PVD, MobileNet identified 59 true positives with no false positives. This model’s high performance underscores its efficiency and robustness in retinal disease classification.

**Table 9:** Confusion matrix for MobileNet.

	Glaucoma	Macula Edema	Normal	PVD
Glaucoma	77	0	1‘	0
Macula Edema	0	39	0	0
Normal	10	0	80	0
PVD	0	0	0	59

Overall, DenseNet121 and MobileNet stand out for their strong and consistent performance across the different retinal conditions, whereas other models exhibit strengths and weaknesses in specific classifications.

### Area Under the curve

**Fig 2:**
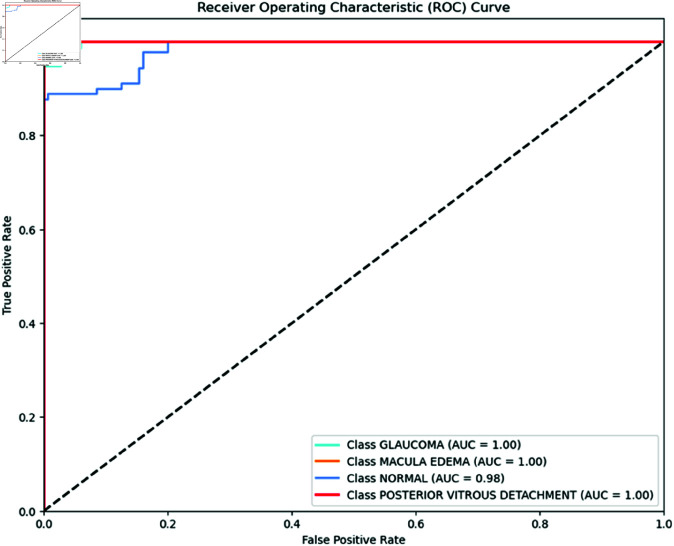
MobileNet ROC Curve per class. MobileNet ROC Curve for each class performs exceptionally well. It achieves an AUC of 1.00 for glaucoma, macular edema, and PVD, indicating perfect performance. The model also shows higher performance with an AUC of 0.98 for normal eye conditions, indicating its effectiveness in distinguishing between normal and diseased states

The Receiver Operating Characteristic (ROC) was adopted to examine the effectiveness of the models. ROC is a probability curve, and AUC represents the degree or measure of separability. The ROC curve is plotted with a true positive rate (TPR) (y-axis) against a false positive rate (FPR) (x-axis). The ROC curve and AUC values are crucial metrics for assessing the performance of classification models across different retinal conditions. MobileNet, as shown in Fig 2, performs exceptionally well with an AUC of 1.00 for glaucoma, macular edema, and PVD. It also shows a high AUC of 0.98 for normal eye conditions, underscoring its effectiveness in distinguishing between normal and diseased states.

**Fig 3:**
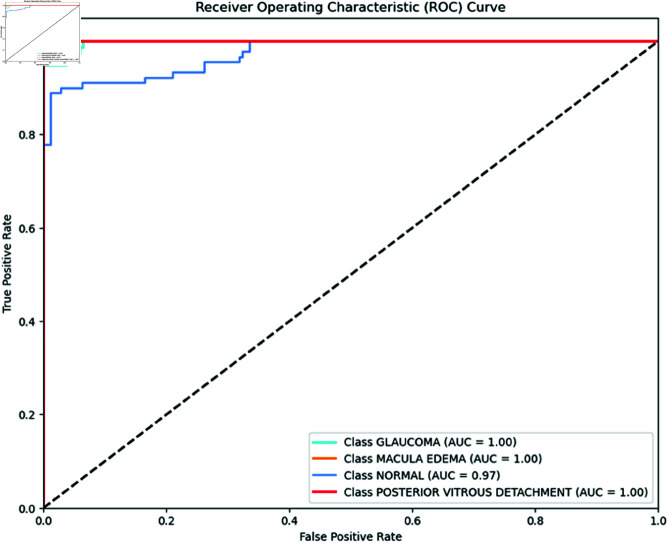
Inception V3 ROC Curve per class. Inception V3 ROC Curve for each class shows impressive results. It achieves an AUC of 1.00 for glaucoma, macular edema, and PVD, indicating perfect performance. The model also demonstrates high performance with an AUC of 0.97 for normal eye conditions, indicating its strong capability in classifying healthy eyes effectively.

Moreover, the Inception V3 model, as illustrated in Fig 3, achieves an AUC of 1.00 for glaucoma, macular edema, and PVD. The model also demonstrates high performance with an AUC of 0.97 for normal eye conditions, indicating its strong capability in classifying healthy eyes effectively.

**Fig 4:**
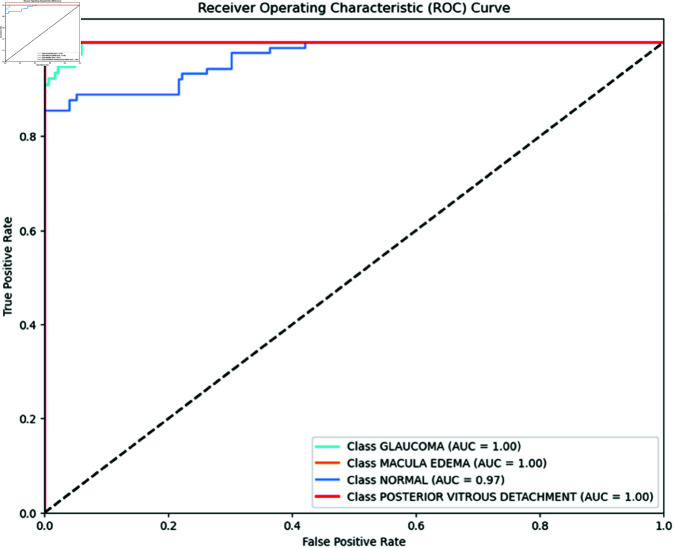
DenseNet121 ROC Curve per class. DenseNet121 ROC Curve for each class shows impressive results. It achieves an AUC of 1.00 for glaucoma, macular edema, and PVD, indicating perfect performance. The model also performs strongly with an AUC of 0.97 for normal eye conditions.

Fig 4 presents the ROC curves for the DenseNet121 model, illustrating its classification performance for each condition. The AUC values indicate the model’s ability to distinguish between each class and the others. For DenseNet121, the AUC values are depicted, showing that this model also excels with an AUC of 1.00 for glaucoma, macular edema, and PVD. DenseNet121 demonstrates a high AUC of 0.90 for normal eye conditions, reflecting its effective performance in distinguishing normal cases from pathological conditions.

**Fig 5:**
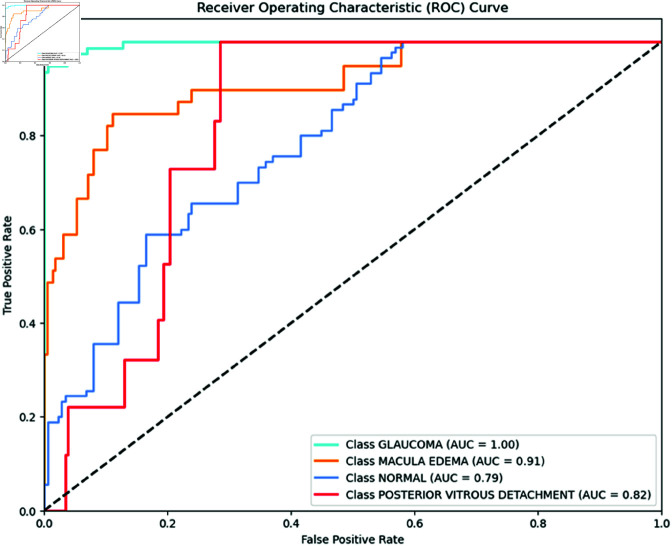
ResNet50 ROC Curve per class. ResNet50 ROC Curve for each class achieves an AUC of 1.00 for glaucoma and 0.91 for macular edema, indicating strong performance. However, it struggles to identify normal cases (AUC=0.79) and PVD (AUC=0.82).

In Fig 5, ResNet50 model performs strongly in classifying glaucoma and macular edema, achieving high recall and precision. However, it struggles with identifying normal cases and PVD, resulting in several misclassifications. The high AUC scores suggest that with additional tuning and possibly more training data, ResNet50’s performance could be further enhanced. The overall evaluation underscores the effectiveness of MobileNet, DenseNet121, and Inception V3 in retinal disease classification, highlighting their robustness and reliability.

### Model accuracy versus model loss per Epoch

**Fig 6:**
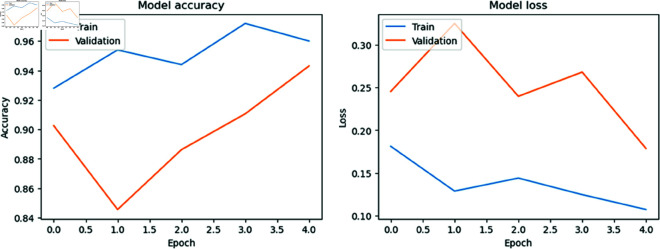
Model accuracy vs model loss per Epoch for MobileNet.

**Fig 7:**
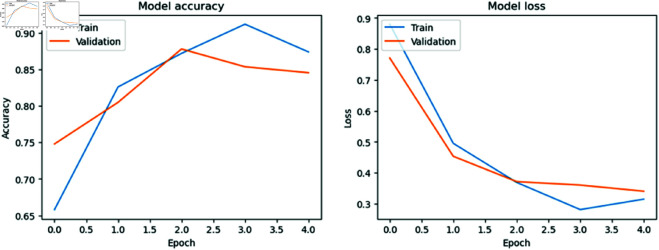
Model accuracy vs model loss per Epoch for Inception V3.

**Fig 8:**
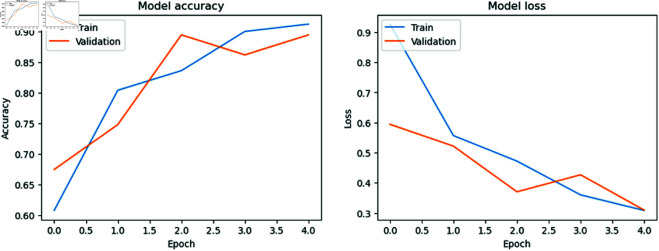
Model accuracy vs model loss per Epoch for DenseNet121.

**Fig 9:**
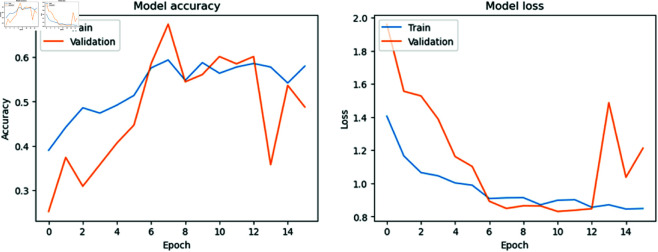
Model accuracy vs model loss per Epoch for ResNet50.

Figs 6, 7, 8, and 9 show the training and validation accuracies and losses for the CNN models with each epoch. We mention that the difference between training and validation accuracy indicates a potential issue with overfitting. Despite using augmentation techniques such as rotation, flipping, and translation, the validation loss remains inconsistent, suggesting a need for further optimization. However, it is reassuring to note that MobileNet, DenseNet121, and Inception V3 generally improve with each epoch, demonstrating ongoing enhancement throughout the training process. This underscores the robustness of the MobileNet, DenseNet121, and Inception V3 models and their capacity to generalize to unseen data, affirming their reliability and suitability for real-world applications.

## Interpretability of the CNN models

To gain deeper insights into the decision-making process of our CNN model, we employed interpretability techniques such as Grad-CAM and saliency maps, following a similar approach as outlined in [49,50]. These visualization methods allowed us to highlight specific regions of the input images that significantly influenced the model’s predictions, thereby enhancing the transparency and interpretability of the classification process.

**Fig 10:**
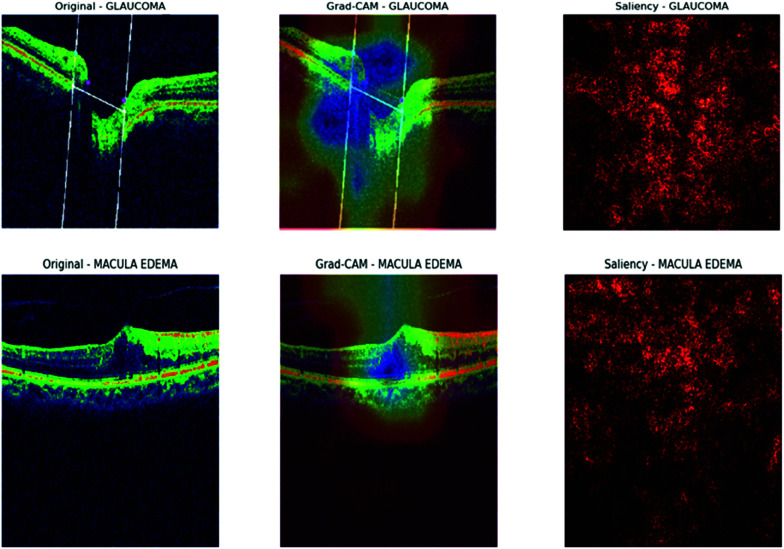
Gradcam and saliency map for MobileNet.

**Fig 11:**
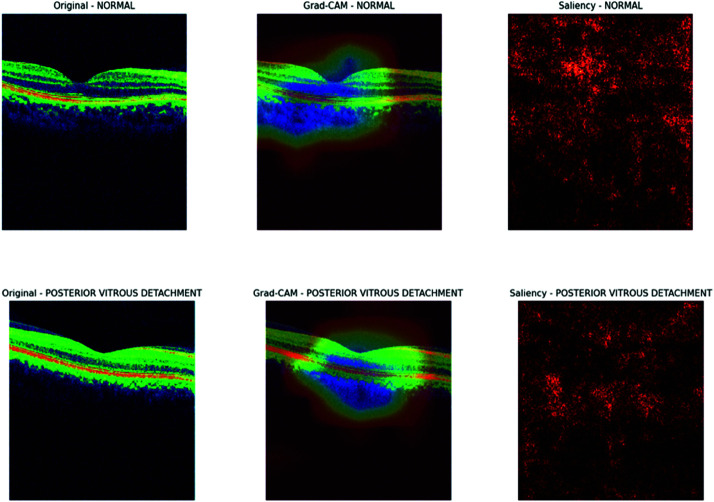
Gradcam and saliency map for MobileNet.

As shown in Figs 10 and 11, the Grad-CAM visualizations (bottom row) consistently focus on the optic nerve head and surrounding retinal nerve fiber layer, which are clinically significant areas associated with glaucomatous damage. This consistent focus across multiple convolutional layers suggests that the model has successfully learned relevant spatial features. Meanwhile, the saliency maps (top-right of each pair) highlight the regions with the most influence on the model’s output. Although saliency maps can sometimes appear noisy, they consistently demonstrate strong activation near the central disc area of the retina, reinforcing the results of Grad-CAM. The combination of these techniques confirms that the model does not base its predictions on random or irrelevant image features. Instead, it aligns its focus with known pathological regions, instilling confidence in the model’s interpretability and potential deployment in clinical decision-support systems.

**Fig 12:**
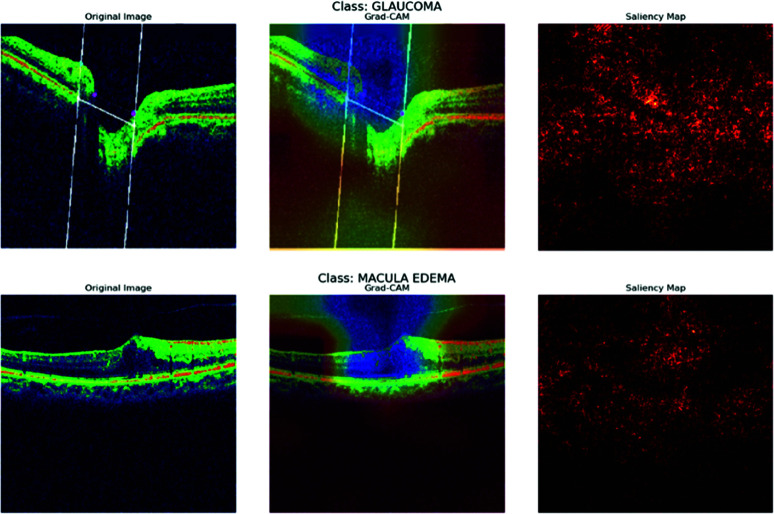
Gradcam and saliency map for DenseNet121.

**Fig 13:**
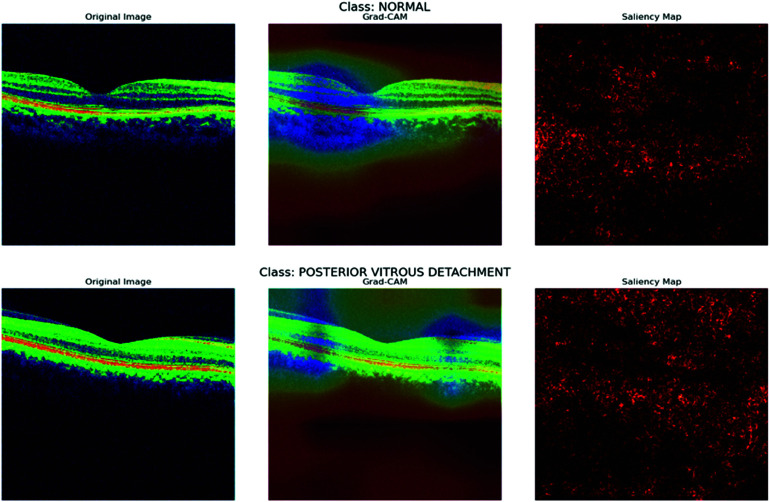
Gradcam and saliency map for DenseNet121.

The visualization results of the DenseNet121 model, as shown in Figs 12 and 13, provide valuable insights into the model’s decision-making process for OCT image classification. In the Grad-CAM heatmaps, the colors represent the importance of each region in the image for the model’s prediction. Red indicates high importance, yellow indicates medium importance, green indicates low importance, and blue indicates very low importance. The model focuses on the optic disc and surrounding retinal tissue for glaucoma images, assigning high importance (red) to regions showing damage, consistent with clinical diagnoses. Similarly, the model emphasizes the macular region for macular edema images, assigning high importance (red) to areas affected by edema. In normal images, the model focuses on the overall retinal structure, including the optic disc and macular region, with low importance (green) assigned to most areas. For images of posterior vitreous detachment, the model directs attention to the vitreous body and retina, assigning high importance (red) to regions exhibiting detachment. These findings demonstrate the potential of deep learning models for accurate and efficient diagnosis of retinal diseases from OCT images, fostering optimism about the model’s application in clinical settings.

**Fig 14:**
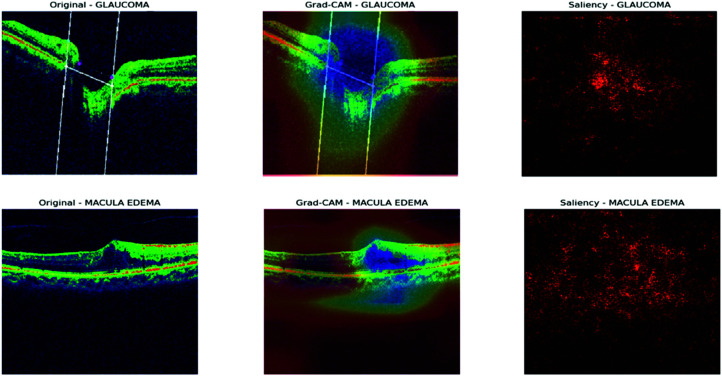
Gradcam and saliency map for Inception V3.

**Fig 15:**
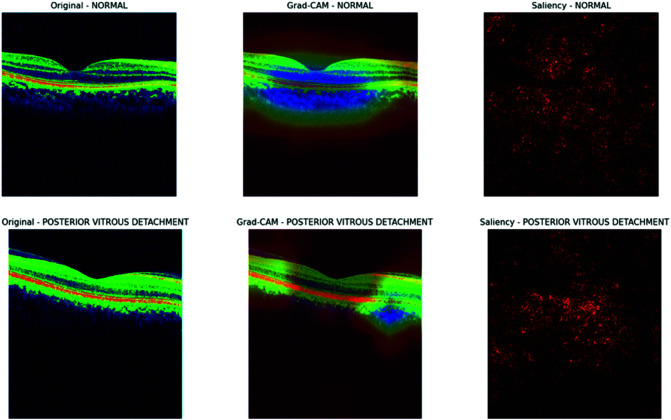
Grad-cam and saliency map for Inception V3.

The visualization results of the Inception V3 model, as presented in Figs 14 and 15, also provide valuable insights into the model’s decision-making process for OCT image classification. The Grad-CAM heatmap focuses on the optic disc and surrounding retinal tissue for glaucoma, indicating that the model is searching for signs of damage or abnormalities in this region. In contrast, the saliency map shows a more scattered attention pattern. In the case of macular edema, the Grad-CAM heatmap highlights the macular region, suggesting the model is detecting fluid accumulation or thickening in this area. The normal category exhibits a relatively even distribution of attention across the retinal layers in the Grad-CAM heatmap, indicating that the model is looking for a typical retinal structure, while the saliency map displays a more random attention pattern. The Grad-CAM heatmap focuses on the vitreous body and retina for posterior vitreous detachment, suggesting that the model identifies signs of detachment or abnormalities at the vitreoretinal interface. Overall, the Grad-CAM and saliency maps provide crucial insights into the Inception V3 model’s decision-making process for OCT image classification. By analyzing these visualizations, we can better understand how the model utilizes different regions of the image to make predictions. This information can be leveraged to improve the model’s performance, interpretability, and reliability in clinical settings.

**Fig 16:**
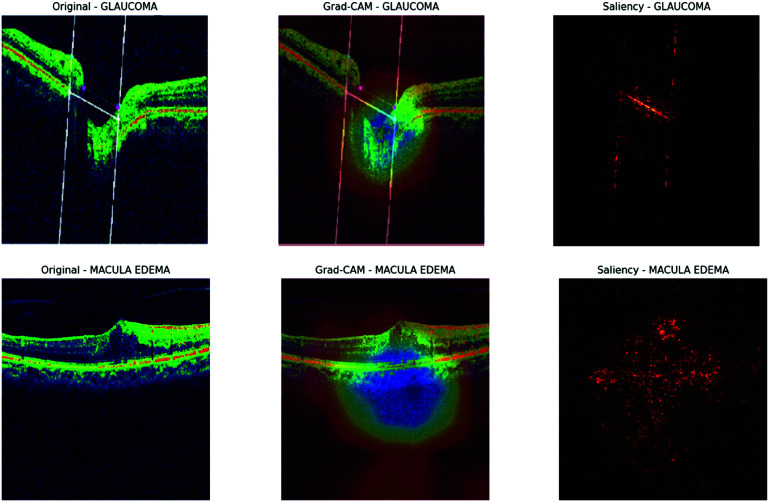
Grad-cam and saliency map for ResNet50.

**Fig 17:**
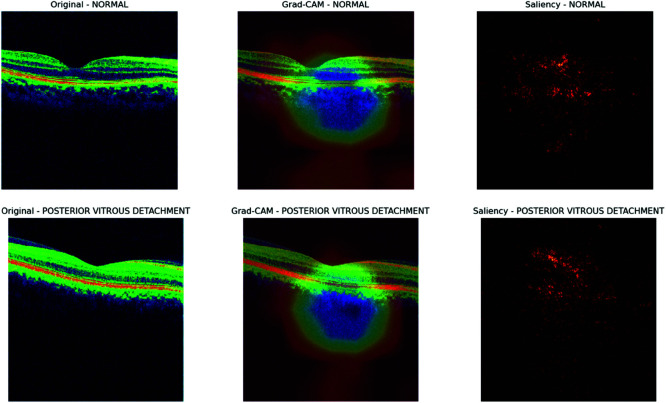
Grad-cam and saliency map for ResNet50.

The visualization results of the ResNet50 model, as shown in Figs 16 and 17, provide valuable insights into the model’s decision-making process for classifying OCT images. In the Grad-CAM heatmaps, the colors indicate the importance of different regions within the image for the model’s predictions: red signifies high importance, yellow indicates medium importance, green represents low importance, and blue corresponds to very low importance.

For images depicting Glaucoma, the model concentrates on the optic disc and the surrounding retinal tissue, assigning high importance (red) to areas displaying damage, which aligns with clinical diagnoses. In the case of Macular Edema images, the model focuses on the macular region and assigns high importance (red) to areas affected by edema. For Normal images, the model evaluates the overall retinal structure, including the optic disc and macular region, but assigns low importance (green) to most areas. In images of Posterior Vitreous Detachment, the model draws attention to the vitreous body and retina, similarly assigning high importance (red) to areas showing detachment.

These findings illustrate the potential of deep learning models for the accurate and efficient diagnosis of retinal diseases through OCT images and underscore the significance of visualization techniques in comprehending the model’s decision-making process.

## Discussion

In this study, we developed and compared the performance of various modernized CNN models—MobileNet, DenseNet121, Inception V3, and ResNet50—for the detection and classification of retinal diseases, including glaucoma, macular edema, PVD, and normal cases in Ghana. We utilized Bayesian Optimization for hyperparameter tuning. This approach allows for systematically and efficiently exploring the hyperparameter space, surpassing traditional methods like random search [30,51]. It enhances model performance and ensures consistency across different architectures, thereby establishing a robust foundation for the classification of retinal diseases and instilling confidence in the reliability of our results.

The findings related to the first research question—how modern CNN models (Inception V3, DenseNet121, MobileNet, and ResNet50) enhanced with Gaussian Process-based Bayesian Optimization effectively detect and classify multiple retinal diseases—revealed that MobileNet and DenseNet121 outperformed other CNN models in retinal disease detection and classification, with MobileNet emerging as the most efficient model. MobileNet’s superior accuracy and balanced performance across all evaluated diseases demonstrate its efficiency and versatility. This aligns with previous findings highlighting MobileNet’s lightweight architecture and suitability for practical applications [52]. The high precision, recall values, and consistently high AUC value underscore MobileNet’s robustness in classifying various retinal conditions. A previous study [53] has reported similar findings, reinforcing MobileNet’s versatility and efficiency in managing complex retinal disease datasets and achieving high performance in real-world scenarios. Both studies accentuate MobileNet’s practicality for large-scale deployment in healthcare.

Additionally, the findings related to the second research question—how well pre-trained models perform in retinal disease-specific cases—indicated that MobileNet performed exceptionally compared to other CNN models. It achieved perfect precision, recall, and F1-score in detecting macular edema and PVD. This is a significant finding, as a previous study [54] has primarily focused on classifying other diseases, such as diabetic retinopathy, using MobileNet without highlighting its effectiveness in detecting macular edema. This emphasizes MobileNet’s vast potential in addressing critical medical challenges, encouraging further research and application. For glaucoma detection, our results surpassed those of previous findings, which utilized Pulsed Coupled Neural Networks and multilayer back propagation neural networks [15]. Notably, Inception V3’s ability to achieve high precision and recall in glaucoma detection and classification highlights its effectiveness in scenarios where detailed feature extraction is essential, aligning with its architectural strengths in capturing multi-scale features [55]. DenseNet121 demonstrated commendable performance in classifying normal eye conditions and PVD, reflecting its effective feature extraction capabilities and dense connectivity. These findings support the model’s ability to maintain gradient flow and capture intricate features, contributing to its high precision and recall. DenseNet121’s performance is consistent with previous studies [56,57], underscoring its strengths in handling diverse image characteristics. Although ResNet50 is a deep and sophisticated model, we observed that it struggled to accurately classify normal eye conditions and macular edema. This difficulty may have arisen from potential overfitting or distinguishing subtle differences between classes. This suggests that deeper models may need further adjustments or more focused training data to improve performance.

### Comparison with other models

Table 10 compares our models with others from the literature that address retinal diseases such as DME, choroidal neovascularization (CNV), and AMD. Although direct comparisons are inherently challenging due to differences in disease types, datasets, and methodologies, we can draw several key observations:

**Table 10:** Comparison of various models for retinal disease classification.

Author	Dataset	Class	Method	Accuracy (%)
[58]	MENDELY dataset	CNV, DMD, DME, Normal	Deep CNN (4 CL + 4 Max Pooling + 2 FL)	97.01%
[59]	Bangladesh	cataract, chalazion, and squint.	VGG16	95.90%
			VGG19	94.20%
			MobileNet	97.49%
			Xception	95.10%
			Inception V3	95.33%
			DenseNet121	96.92%
[60]	Prasad Eye Institute, based in Hyderabad, India.	Glaucoma	Deep CNN model with convolutional layers (four convolution layers, four max pooling layers) and two fully connected layers.	93.75%
[61]	Kaggle	Glaucoma	ConvNeXt Base with a Convolutional Block Attention Module	82.16%
[62]	ODIR	Diabetic Retinopathy	Inception-ResNet V2	97.5%
[63]	Indian dataset	Glaucoma	Ensemble model of ResNet50, VGG16) and Random Forest	95.41%
[64]	Kaggle	Diabetic retinopathy	Xception	83.7%
[65]	Not stated	CNV, DME, DME	CNN	95.30%
[66]	Kaggle website	Diabetic retinopathy	Xception	80%
[67]	Kaggle.com	Diabetic retinopathy	DenseNet121, ResNet50	85%
[68]	APTOS 2019 Blindness Detection dataset	Diabetic Retinopathy	HDR-EfficientNet model	98%
Proposed	Watborg Eye Clinic (Ghana Dataset)	Glaucoma, PVD, Macula Edema, Normal		
			MobileNet	96.00
			Inception V3	83.00
			DenseNet121	95.00
			ResNet50	61.00

**General performance:** Our models showed robust performance, particularly after fine-tuning. MobileNet achieved the highest performance in our study, with an accuracy of 96% and an AUC of 99%. This result is comparable to the performance reported in a prior study such those in [58] using datasets like MENDELY, which achieved 97.01% accuracy for CNV, drusen macular degeneration (DMD) and DME classification. DenseNet121 is closely followed, with an accuracy of 95% and the same AUC score, highlighting its effectiveness in classifying retinal diseases. Other literature also showcases high-performing models. For example, studies such as [59] compared VGG16 (95.90%), MobileNet (97.49%), and DenseNet121 (96.92%) on the Bangladesh dataset, demonstrating MobileNet’s consistent superiority.

**Disease-specific accuracy:** The classification performance varied across different diseases, reflecting the unique characteristics of each retinal condition. For example, diseases like DME or CNV exhibit distinct image features that can lead to different performance metrics compared to conditions such as glaucoma or macular edema. In our study, MobileNet demonstrated the highest overall accuracy, while DenseNet121 and Inception V3 showed strong performance in glaucoma classification, both achieving 95% accuracy, underscoring their relevance for specific conditions. This performance pattern underscores the importance of disease-specific image features in model performance and surpasses a study by [60] that utilized a CNN model with four convolutional and max pooling layers alongside two fully connected layers for glaucoma classification based on data from the Prasad Eye Institute in India, achieving 93.75% accuracy. It also surpasses [61], which used ConvNeXt Base with a convolutional block attention module on Kaggle’s glaucoma dataset and obtained a lower performance of 82.16%

**Model robustness:** Our findings indicated that models such as MobileNet, DenseNet121, and Inception V3 demonstrated strong robustness, achieving consistent accuracy across different disease types. Notably, MobileNet achieved the best overall performance, with an F1-score of 97%, indicating an excellent balance between precision and recall. This performance aligns with, and sometimes surpasses, previous research findings. For instance, one study reported an F1-score of 94.91% for cataract and squint classification using similar architectures [59], while MobileNet in our study exceeded that across all performance indicators.

**Methodological insights:** The application of transfer learning and fine-tuning was essential in enhancing model performance, allowing pre-trained models to adapt effectively to new disease types. Moreover, employing Bayesian optimization for hyperparameter tuning proved a significant advantage, further improving model performance and consistency. While Adam and RMSprop optimizers were used in a previous study [59], our approach using Bayesian optimization resulted in consistently higher accuracy and more reliable outcomes.

**Unique contributions:** Our study makes several novel contributions that distinguish it from existing research. First, we utilized a localized dataset from Ghana, a population often underrepresented in retinal disease studies. Unlike other studies [62–68], this localized focus is crucial for developing AI models tailored to the needs of resource-limited settings, particularly in many parts of Africa. Second, we implemented Bayesian optimization for hyperparameter tuning, providing a more systematic and efficient approach than the random search method commonly employed in other studies. As a result, DensNet121 in our study achieved an F1-score of 95%, compared to 93.8% in a previous study [59].

Implementing CNN models like MobileNet and DenseNet121 presents significant opportunities for real-world applications in medical settings. These models can be utilized for various medical purposes, including the early detection and classification of retinal diseases. They can also be integrated into mobile applications, providing healthcare practitioners in resource-limited environments with rapid and accurate diagnostic tools. By enabling timely interventions, these tools can help reduce the burden of retinal diseases and improve patient outcomes. The potential of these models to make healthcare more accessible to underserved populations should instill a sense of hope and optimism. Lightweight CNN models like MobileNet are particularly well-suited for deployment on simple devices. However, to enhance their adoption and utility in clinical settings, it is crucial to train healthcare providers who may not be familiar with AI models, enabling them to use these tools effectively.

While this study demonstrates promising results using modern CNN architectures and Bayesian optimization for classifying retinal diseases, real-world clinical implementation presents several challenges. These challenges include integration into existing hospital workflows, variability in imaging devices across facilities, and the need for real-time processing capabilities. Additionally, clinician acceptance may be hindered by concerns regarding model interpretability and trust in automated systems. Infrastructure limitations, particularly in low-resource settings, may also affect the deployment of these AI tools. Furthermore, beyond data anonymization, it is worth noting that deploying AI models in clinical practice raises several ethical concerns that policymakers must carefully address before widespread adoption. One key issue is algorithmic bias, where models trained on data not representative of diverse patient populations may produce inaccurate or unfair predictions across different subgroups, including age, ethnicity, or gender. This can lead to disparities in diagnosis or treatment recommendations. Transparency and explainability are critical; clinicians must understand and trust model outputs, especially in high-stakes decisions. Additionally, accountability must be clearly defined—there should be clarity regarding who is responsible for system errors. The informed consent process may evolve, particularly if AI-driven decisions are to be integrated into the healthcare system. Ensuring that patients are aware of and understand the role of AI in their treatment is vital for maintaining ethical integrity.

These considerations highlight the need for robust clinical validation, interdisciplinary collaboration, and continuous monitoring of model performance in real-world settings. The need for continuous monitoring of model performance should reassure the audience about the safety and effectiveness of AI in healthcare. Future efforts will focus on addressing these challenges by actively involving clinicians in the model development process, piloting deployments within healthcare facilities, and implementing feedback mechanisms to ensure continuous refinement of the models to align them with clinical needs.

### Future directions

While the results are promising, several potential research avenues could improve model performance and applicability:

**Expanding the dataset**: With its high-quality clinical images, the dataset is a valuable resource for assessing deep learning models in real-world scenarios. However, Ghana has a data scarcity issue and the high costs associated with obtaining a representative sample nationwide. As a result, we had to rely on data from a single health facility, which may have introduced bias into our study. To enhance the robustness and generalizability of the models, future research should include datasets from multiple eye care centers across various geographic locations in Ghana.

**Integrating multi-modal data**: Another promising avenue for future research is the integration of multi-modal data. By exploring the combination of OCT images, fundus photographs, and patient demographic data, researchers could enhance the robustness and accuracy of classification models—opening up new frontiers in the field of medical imaging and machine learning.

**Hybrid models**: Hybrid models represent another area of potential innovation. Investigating the use of ensemble learning or hybrid architectures could leverage the strengths of multiple models to achieve higher accuracy and better generalization, thereby pushing the boundaries of what is possible in machine learning for medical imaging.

**Clinical validation**: As previously mentioned, our study has shown promising results in automated retinal disease classification using CNN-based models. However, the transition from research to real-world clinical implementation requires further steps. One crucial area for future research is structured clinical validation. This process is vital as it ensures the model’s reliability, interpretability, and generalizability in practical healthcare settings. To achieve this, we propose a comprehensive multi-phase clinical validation framework. The first phase would involve retrospective testing of the model on annotated clinical datasets sourced from multiple institutions. This testing aims to evaluate the model’s performance consistency across diverse populations and imaging protocols. The second phase would be a prospective clinical study, where the model’s predictions are compared against the diagnoses made by ophthalmologists in real-time. This comparison benchmarks the model’s performance against human experts and uncovers edge cases and clinical scenarios where the model may need further tuning. Our proposed validation framework, inspired by prior studies that validated deep learning models for OCT interpretation through clinical simulations and comparisons with retinal specialists [69], validated a diabetic retinopathy model across multiple eye hospitals in India [70] and implemented AI-based retinal screening tools in real clinical workflows [71], holds great promise for the future of AI in ophthalmology. These studies highlight that robust clinical validation is essential for translating AI models into safe, effective, and ethical tools for ophthalmic practice. The third phase would involve longitudinal validation, in which the model is integrated into a clinical decision support system (CDSS). This phase would evaluate the model’s impact on patient outcomes, workflow efficiency, and diagnostic accuracy over time. To enhance adaptability and trust, real-time monitoring can be complemented by clinician feedback and model retraining loops.

**Exploring other methodologies**: While we have employed techniques such as Bayesian optimization, data augmentation, dropout, and early stopping to mitigate overfitting and underfitting, traces of these issues persist. Therefore, it is clear that future work will need to focus on further improving the generalizability and robustness of the model through the following steps:

1. Enhancing Bayesian Optimization: Future studies will refine the search space and incorporate multi-objective Bayesian optimization to balance accuracy and generalization simultaneously. This may also include optimizing data augmentation strategies and regularization parameters alongside model architecture.
2. Advanced Regularization: Incorporating additional regularization techniques such as L1/L2 penalties, label smoothing, and stochastic depth will be explored to control model complexity further and reduce overfitting.
3. Model Ensembling: Ensemble methods (e.g., stacking, bagging) will be applied to combine multiple high-performing models, reducing model variance and improving predictive stability.
4. Domain Adaptation and Fine-Tuning: Future work will explore improved fine-tuning techniques for transfer learning, especially when adapting to new imaging devices or populations, to enhance generalization and reduce underfitting in unseen domains.

## Conclusion

This study examined various CNN models—specifically DenseNet121, MobileNet, Inception V3, and ResNet50—combined with Gaussian process-based Bayesian optimization to detect and classify multiple retinal diseases. Among all the evaluated CNN models, MobileNet and DenseNet121 demonstrated a strong ability to accurately detect and classify various retinal conditions, including glaucoma, macular edema, PVD, and healthy eye cases. Notably, except for ResNet50, the other CNN models achieved higher accuracy than other state-of-the-art deep learning methods used for classification. Both MobileNet and DenseNet121 showed significant promise for deployment in clinical settings, raising optimism about medical technology’s future. Their precise classification of multiple retinal diseases can aid healthcare professionals in making timely and informed decisions. Additionally, there is a pressing need to develop a mobile application that integrates these trained models, enabling healthcare practitioners to quickly and accurately diagnose retinal diseases. This would significantly enhance access to advanced retinal diagnostics in clinical environments, particularly in regions with limited medical resources.
